# Comparison of Heart Rate Monitoring Accuracy between Chest Strap and Vest during Physical Training and Implications on Training Decisions

**DOI:** 10.3390/s21248411

**Published:** 2021-12-16

**Authors:** Jakub Parak, Mikko Salonen, Tero Myllymäki, Ilkka Korhonen

**Affiliations:** 1 Firstbeat Technologies Oy, 40100 Jyväskylä, Finland; mikko.salonen@firstbeat.com (M.S.); tero.myllymaki@firstbeat.com (T.M.); ilkka.korhonen@firstbeat.com (I.K.); 2 Faculty of Medicine and Health Technology, Tampere University, 33720 Tampere, Finland

**Keywords:** beat-to-beat detection, heart rate, chest strap, vest, sport sensor, Energy Expenditure, Excess Post-exercise Oxygen Consumption, Training Impulse

## Abstract

Heart rate (HR) and heart rate variability (HRV) based physiological metrics such as Excess Post-exercise Oxygen Consumption (EPOC), Energy Expenditure (EE), and Training Impulse (TRIMP) are widely utilized in coaching to monitor and optimize an athlete’s training load. Chest straps, and recently also dry electrodes integrated to special sports vests, are used to monitor HR during sports. Mechanical design, placement of electrodes, and ergonomics of the sensor affect the measured signal quality and artefacts. To evaluate the impact of the sensor mechanical design on the accuracy of the HR/HRV and further on to estimation of EPOC, EE, and TRIMP, we recorded HR and HRV from a chest strap and a vest with the same ECG sensor during supervised exercise protocol. A 3-lead clinical Holter ECG was used as a reference. Twenty-five healthy subjects (six females) participated. Mean absolute percentage error (MAPE) for HR was 0.76% with chest strap and 3.32% with vest. MAPE was 1.70% vs. 6.73% for EE, 0.38% vs. 8.99% for TRIMP and 3.90% vs. 54.15% for EPOC with chest strap and vest, respectively. Results suggest superior accuracy of chest strap over vest for HR and physiological metrics monitoring during sports.

## 1. Introduction

Monitoring of heart rate (HR) and its variability (HRV) during athletic training and rest provides critical insights on the impact of training on the athlete’s physiology. Utilizing HR and HRV data helps to optimize training load and recovery for optimal development of performance and for preventing injuries. Key metrics for training load monitoring include Training Impulse (TRIMP) [1], Excess Post-exercise Oxygen Consumption (EPOC) [2], and Energy Expenditure (EE). These training parameters can be estimated with reasonable accuracy from training HR data [2,3,4,5]. Accurate monitoring of beat-by-beat HR data is a requirement for reliable estimation of an athlete’s physiology and for making appropriate data-driven coaching decisions [6,7].

Chest straps are widely used for HR monitoring during athletic training. The high HR measurement accuracy, low bias and error rate of chest strap HR monitors have already been examined and demonstrated against clinical ECG [8,9]. However, their usage, comfort and stability may be challenging [10]. Hence, vests with integrated electrodes for HR monitoring have been introduced to overcome the shortcomings of chest straps. However, their accuracy as compared to chest straps has been questioned [11,12].

The aim of this study was to compare the accuracy of a chest strap and a vest against a gold standard reference (clinical ECG monitor) for HR and HRV monitoring, and to analyze the impact of their accuracy into accumulated physiological metrics TRIMP, EPOC and EE used by coaches and athletes for training monitoring and planning.

## 2. Materials and Methods

### 2.1. Subjects and Study Protocol

Twenty-five healthy non-smoking volunteers (six females) without any cardiovascular conditions having regular weekly exercising habits participated to the study (see Table 1). The study followed the Helsinki declaration for study ethics. A written informed consent was obtained from the participants, and they were free to discontinue the study at any point.

The subjects performed twice an exercise protocol outdoors including jogging at ~8 km/h (10 min), running stairs up and down (1 min), Nordic walking (4 min), upper body exercise (touch toes and hands up; 1 min) and upper body rotation with stick on shoulders (1 min), totaling ~20 min. The exercise was guided and controlled by a researcher. Data collection was conducted in two parts during spring and summer 2021. During the first, early spring period, the outdoor temperature was between 4–12 °C, the terrain was not slippery, and it did not rain. During the second period, early summer, outdoor temperature was between 17–27 °C.

### 2.2. Data Aqusition

**Figure 1:**
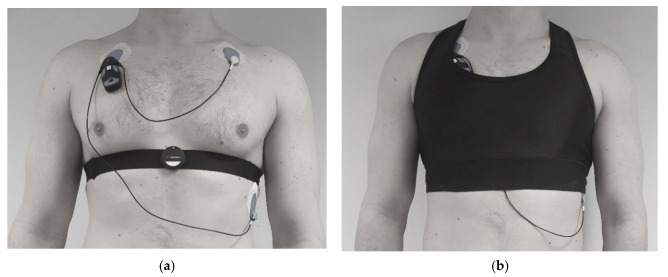
Sensor placement on body: (**a**) ECG sensor attached to a chest strap and the reference device; (**b**) Vest and the reference device. ECG sensor was attached to the vest on the back side.

Single-lead ECG signals at 125 Hz were collected with Suunto Movesense sensor (Suunto Oy, Vantaa, Finland) connected to either a standard chest strap (Suunto Oy, Vantaa, Finland) or a widely used heart rate vest (Bromely Sports, Sialkot, Pakistan). ECG data were transmitted from the sensor in real-time over Bluetooth and logged to mobile phone placed in the subject pocket during the protocol. The reference ECG signal at 256 Hz was measured with a 3-lead medical grade Holter ECG device (Bittium Faros, Bittium, Finland). The subjects wore the chest strap and the vest as recommended by their manufacturer, including proper moistening of the electrode areas and correct fit for subjects of various body sizes. To avoid any mechanical interference between the chest strap and the vest electrodes, the subjects wore only one study device (chest strap or vest) at a time and repeated the protocol twice, in randomized order. Location of the chest strap, the vest, and the reference device placement is shown in Figure 1.

### 2.3. Signal Processing

The reference RR-intervals (RRI) were derived with an algorithm implemented in the reference device. The RRI were extracted from the raw ECG data of chest strap and vest by using the same automatic proprietary R-peak detection algorithm for both (Firstbeat Technologies Oy, Jyväskylä, Finland). The R-Peak detection algorithm has been derived from Pan-Tompkins methodology and further optimized for use in sports sensors. The grade of artifactual RRIs were estimated with artifact correction method detecting artefacts such as extra and missing beats, and movement noise resulting in the corrected data for physiological analysis (Firstbeat Sports SW, Firstbeat, Finland). Recordings with unreliable reference (REF > 15% of detected and corrected artefactual RRIs) were excluded from the analysis. This limit has been used also in previous studies for data exclusion by Föhr et al. [13]. Based on these criteria, 20 and 22 measurements were included for final analyses with chest strap and vest, respectively.

### 2.4. Estimation of the Measurement Error

First, HR and RRI measurement accuracies were calculated as suggested by Parak et al. [14] including time synchronization of the target and the reference signals, pairing each reference and tested device RRI, identifying extra and missing beats, and calculating error metrics for paired intervals. HR performance metrics were estimated in 5 s successive windows [15]. Mean absolute error (MAE), mean absolute percentage error (MAPE), accuracy (100%—MAPE) and reliability (% of time that the absolute error is smaller than 5 bpm) were estimated for HR. Thereafter, EE, TRIMP, EPOC and average HR were estimated for reference device, chest strap and vest with the Firstbeat Sports software (Firstbeat Technologies Oy) as described in [1,2,3]. These methods have been evaluated earlier. The mean error of the EE estimation compared with gold-standard has been reported to be 12.2–13.6 as% coefficient of variation in graded exercise test [4] and the differences between estimated and gold standard EE during the graded exercise test was 0.4–5.7% [16]. For EPOC, strong correlation (r = 0.89) has been reported between EPOC estimated from HR and measured gold-standard EPOC [2]. TRIMP, in turn, is a mathematical equation calculated from HR [1] and hence its accuracy depends directly on the accuracy of HR. After that, MAE, MAPE, and root mean square error (RMSE) were calculated for EE, TRIMP and EPOC for chest strap and vest against the reference data in Microsoft Excel. Finally, Bland–Altman plots were generated for a visual analysis of the error, and bias of HR, RRI, EE, EPOC, and TRIMP.

### 2.5. Statistical Analyzes

The normality of distribution of training parameters datasets was checked by the Shapiro–Wilk test. Pearson correlation coefficient was estimated between normally distributed parameters, while Spearman rank correlation coefficient was applied for the other parameters which were not meeting the normal distribution assumption. The statistical analyzes were performed in R Studio software (1.4.1717).

## 3. Results

### 3.1. Heart Rate Measurement Accuracy

**Figure 2:**
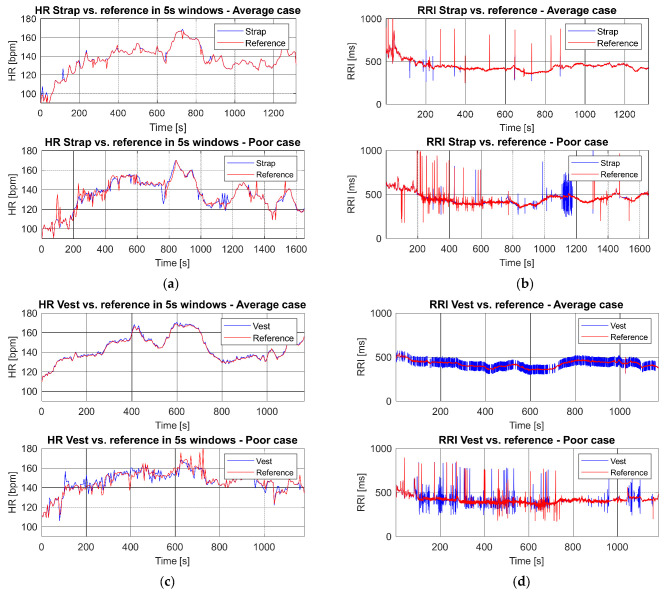
Comparison of HR and RRI from sports sensor and ECG Holter reference during the whole protocol: (**a**) Strap HR poor and average case; (**b**) strap RRI poor and average case; (**c**) vest HR poor and average case; (**d**) vest RRI poor and average case.

**Figure 3:**
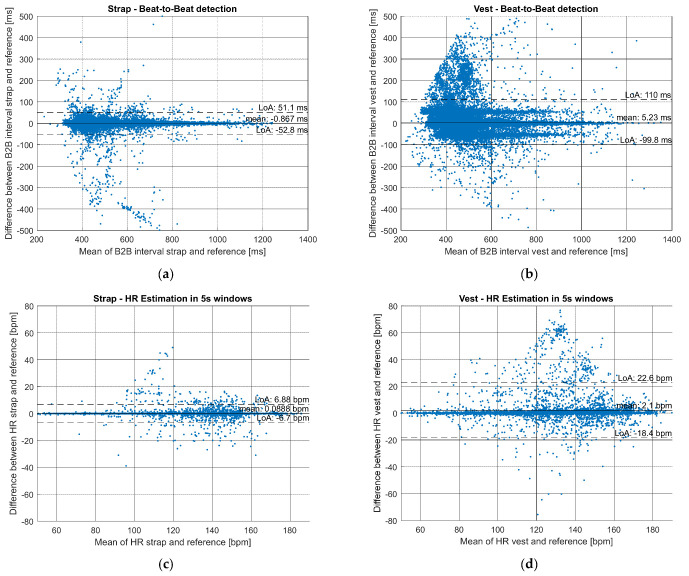
Bland−Altman plot comparing the sensor device and ECG holter reference HR and RRI during entire protocol including all measurements (20 for strap and 22 for vest) (solid horizontal line: bias, dashed lines: 95% confidence limits of agreement): (**a**) RRI strap vs. reference; (**b**) RRI vest vs. reference; (**c**) HR in 5 s windows strap vs. reference; (**d**) HR in 5 s windows vest vs. reference.

The chest strap had a superior accuracy over the vest during sports for both HR (Table 2), RRI (Table 3) and correspondingly also for physiological metrics (Table 4 and Table A1). On average, MAPE for HR during sports was 0.76% for the chest strap and 3.32% for the vest. The vest had a tendency to detect excess number of artefactual beats: only 96.28% of the beats detected with the vest were actual beats while 99.21% of the beats were real heart beats with the chest strap (Table 3). These error percentages may accumulate to significantly wider errors for training parameters (Table 4 and Table A1), especially in some individuals. Examples of an average and a poor case for both HR and RRI monitoring are presented in Figure 2. The increased number of outliers in the vest measurements might be explained by poor or unstable contact of electrodes between skin and vest. Figure 3 presents a Bland–Altman plot of the HR and RRI during the entire protocol from strap and vest compared to clinical ECG reference. The wider LoA and higher bias between the sensor data and reference can be observed in vest measurements.

### 3.2. Training Parameters Estimation Accuracy

**Figure 4:**
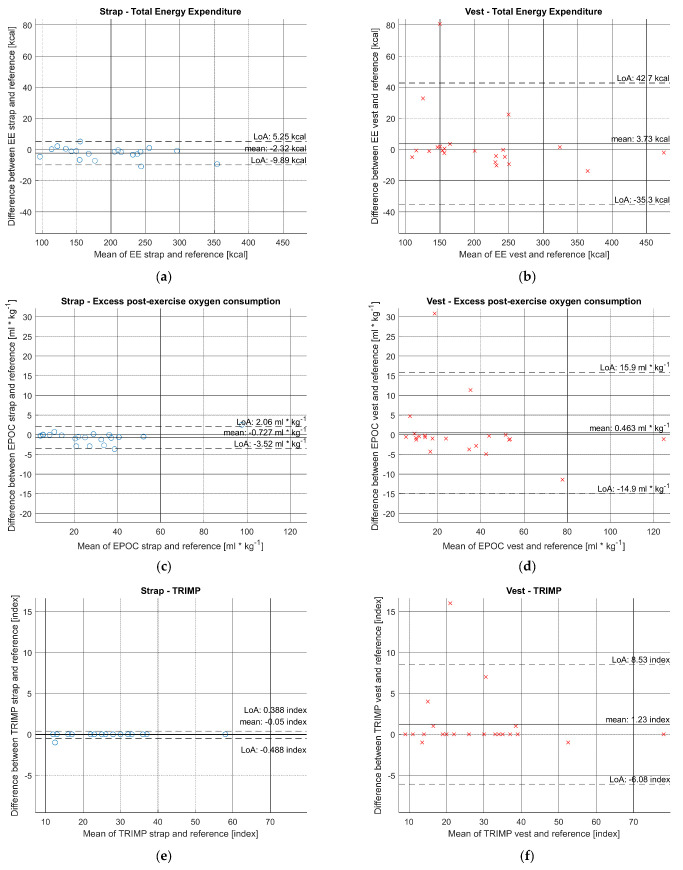
Bland−Altman plot comparing training parameters estimation with using input from sensor device and reference ECG holter during entire protocol (solid horizontal line: bias, dashed lines: 95% confidence limits of agreement): (**a**) Strap—Total Energy Expenditure; (**b**) Vest—Total Energy Expenditure; (**c**) Strap—EPOC; (**d**) Vest—EPOC; (**e**) Strap—TRIMP (**f**) Vest—TRIMP.

The overall summary of error metrics (Table 4) shows better performance of the chest strap compared to the vest. The MAPE range for average HR estimation varied from 0% to 0.83% for the chest strap and from 0% to 23.91% for the vest; for EE from 0.17% to 4.63% for chest strap and from 0.10% to 73.64% for the vest; for TRIMP from 0% to 7.69% for chest strap and from 0% to 123.08% for the vest; and for EPOC from 0.08% to 13.2% for the chest strap and from 0.06% to 935.26% for the vest. The interindividual differences were wide, especially for the vest (Table A1). The higher levels of RMSE error for the vest indicates an increased level of significant outlier error between sensor and reference data. Training parameters estimated using chest strap HR input data had very high correlation (0.99) against vest input HR data (0.89–0.96). Figure 4 shows the Bland-Altman plots of training parameters EE, EPOC and TRIMP. The mean bias levels are highly similar for strap and vest. However, the vest results have much wider LoA, which indicates more outliers in individual measurements.

## 4. Discussion

### 4.1. Principal Findings

We estimated the accuracy of chest strap and vest for monitoring HR during athletic training. We used identical electronics, algorithms, and software to derive HR, HRV and advanced physiological parameters for each sensor type and hence the only difference between the technologies were the form factor of the electrodes and their attachment on the body. The results suggest that the chest strap provides superior HR monitoring accuracy over the vest. The resulting error in advanced physiological training parameters varied widely between the individuals, especially with the vest where the error in advanced training parameters was significantly higher than with the chest strap (e.g., EPOC MAPE 0.38% vs. 8.99% for chest strap and vest, respectively). It appears that, in practice, the HR strap provides reliable results across subjects whereas the HR vest was reliable only for some individuals but not all. These findings challenge the usefulness of the vest-based HR monitoring for assessing training load in athletes.

In the present study, the exercise intensity was mostly light, as indicated by average HR of 123 by the reference device for both sensor tests. Therefore, the study protocol was rather easy and corresponded to a calm exercise in terms physiological demands but also of body movements compared to a typical team sports training session. In terms of heart rate level accuracy, coaches typically monitor the time spent at different HR zones, and especially in the highest training zone (corresponding 90–100% HRmax). The light exercise of the present study resulted to a MAPE of 3.32% and MAE of 3.8 beats per minute for HR with the vest (vs. MAPE of 0.76% and MAE of 0.9 beats per minute with strap). It can be speculated that the similar percentage error level would have resulted in 5–7 beats/min error with the vest when doing high-intensity training at typical HR levels of 171–190 bpm (when HRmax is 190), not even accounting for considerably harder conditions for HR monitoring when the magnitude of body movements is sharply increased. This kind of inaccuracy would have a significant impact on the reported time spent at the 90–100% HR zone, leading to misinterpretations in coaching decisions. 

The beat-to-beat measurement accuracy of the strap in our study was in line with results of the previous studies [8,9]. Poorer performance of the vest sensors in measuring HR has also been reported earlier [11,12]. Our study confirms these findings. Importantly, we aimed to eliminate any other sources of error than the form factor of the electrodes in our study, and hence the difference in performance found in our study may be assigned to the difference in the sensor mechanical design and fit on body. 

The measurement error in HR and HRV accumulates while estimating advanced physiological metrics. Regarding TRIMP, the MAPE was 0.38% with the chest strap and 8.99% with the vest. For comparison, in a typical soccer game the accumulated TRIMP values are between 250–300 units. In practice, this means that MAPE for the vest would lead to error of around 25–30 units in TRIMP. TRIMP over several training sessions are also used for assessing acute and chronic training load levels (summing 7d and 28d TRIMP, respectively) and their ratio. These parameters are widely used as a basis for training planning and injury prevention, and the high measurement error with vest is likely to further reduce the usefulness of such data.

The reasons behind the inaccuracy of the vest may be speculated. Most likely, the main reason is mechanical artefacts related to upper body movements which easily cause the electrodes to move against the skin. Additionally, the chest strap can more easily be adjusted tightly to different chest circumferences, while vests are typically offered in fixed sizes. The vest sensor is available on a limited number of sizes and is not adjustable and hence may not fit to all body types and sizes. Based on the results of our study, the vest sensor may not perform well for smaller female subjects, or male subjects with bigger upper body proportions. For mid-tall athletes, the results were comparable with chest strap sensor. The difference in reliability is likely to increase in long term usage as maintenance care and adjustments are easier with straps than with vests. The subjective evaluation of the comfort of different form factors was out of scope of this study.

### 4.2. Limitations and Strenghts of the Study 

The study has some strengths and limitations. Based on our literature review, it is the first study providing systematic comparison of HR measurement of chest strap and vest sensors compared to true ECG reference and eliminating other sources of performance difference than the mechanical (electrode) design and form factor. In addition, we studied the impact of the measurement error on the practical training parameters (EE, EPOC, TRIMP) which are widely used in athletic training and derived from measured HR data. The study setup is very realistic, and we tried to control all the parts of the measurement to achieve the most accurate results. 

A weakness in our study is the limited number of subjects and selected population, since the subjects were not professional athletes but recreational trainers. However, the study size was sufficient to show clear differences in the accuracy between the sensors, and this difference would likely not change by widening the population or increasing the size of the study. With professional athletes, it would have been possible to conduct more demanding protocol that would be even more realistic considering typical athletic training, but most probably this would have made the differences in sensor accuracy even higher. Furthermore, we performed the data collection in two parts, and the different outdoor temperatures might affect the clothes of the subjects and contact between sensor and skin. However, the order of the sensor testing was randomized, and hence this should not affect the results systematically.

We also noticed that even if the vest had been designed and is being marketed as unisex, the used model did not fit well for some female subjects. Only one strap and one vest manufacturer’s products were tested in the study. The number of vest manufacturers is still very limited. To our knowledge, the Suunto chest strap product is widely used, accepted, and already tested in several studies in the past. We had to select a product with easy access and possibility to collect raw data to avoid any discrepancies or possible biases between used HR detection and training parameter algorithms.

## 5. Conclusions

In professional sports settings, coaches are balancing the player’s compliance to monitoring and the accuracy of the devices to offer reliable and useful data needed for efficient coaching. This study compared the accuracy of HR and HR-derived physiological metrics between chest strap and vest when the only difference was the electrode form factor. Based on the results, we conclude the accuracy of HR data and HR-derived physiological metrics as collected with the chest strap to be superior to the HR vest when using clinical grade HR monitoring as a reference. The study suggests that HR monitoring with a chest strap provides high-quality data for data-driven coaching decisions in sports settings.

## Figures and Tables

**Table 1:** Subjects’ anthropometric parameters.

Parameter	µ ± σ	Range [Min–Max]
Age (years)	33.88 ± 8.48	[23–58]
Height (cm)	176.36 ± 3.96	[154–193]
Weight (kg))	78.24 ± 16.18	[51–115]
Body mass index (kg/m^2^)	24.99 ± 3.96	[20–39]

**Table 2:** Heart rate error statistics calculated in 5 s windows.

Error Type	Strap	Vest
MAE (bpm)	0.93	3.82
MAPE (%)	0.76	3.32
Reliability (%)	94.61	96.68
Accuracy (%)	99.24	84.70

**Table 3:** Beat-to-beat interval estimation error statistics.

Title 1	Strap	Vest
MAE (ms)	6.54	25.14
MAPE (%)	1.31	5.02
Correct beats (%)	99.21	96.28
Missing beats (%)	0.31	0.96
Extra beats (%)	0.48	2.76

**Table 4:** Training parameters error statistics and statistical analysis results.

Parameter	Error Type	Strap	Vest
Average HR (bpm)	Bias (ME) (bpm)	−0.05	1.77
	MAE (bpm)	0.05	2.23
	MAPE (%)	0.04	2.18
	RMSE (bpm)	0.22	42.99
	Correlation coefficient	r = 0.99, (*p* < 0.001) (^2^Pe)	r = 0.96, (*p* < 0.001) (^2^Pe)
Energy Expenditure, Total (kcal)	Bias (ME) (kcal)	−2.32	3.73
	MAE (kcal)	3.22	9.45
	MAPE (%)	1.70	6.63
	RMSE (kcal)	4.42	19.80
	Correlation coefficient	r = 0.99, (*p* < 0.001) (^2^Pe)	ρ = 0.93, (*p* < 0.001) (^1^Sp)
EPOC Peak (ml/kg)	Bias (ME) ((mL/kg)	−0.73	0.46
	MAE ((mL/kg)	1.08	3.82
	MAPE (%)	3.90	54.15
	RMSE (mL/kg)	1.57	7.69
	Correlation coefficient	ρ = 0.99, (*p* < 0.001) (^1^Sp)	ρ = 0.89, (*p* < 0.001) (^1^Sp)
TRIMP (index)	Bias (ME) (index)	−0.05	1.23
	MAE (index)	0.05	1.41
	MAPE (%)	0.38	8.99
	RMSE	0.01	3.84
	Correlation coefficient	ρ = 0.99, (*p* < 0.001) (^1^Sp)	ρ = 0.94, (*p* < 0.001) (^1^Sp)

^1^Sp—Spearman correlation coefficient. ^2^Pe—Pearson correlation coefficient.

## Appendix A

**Table A1:** Training parameters detailed results, MAE and MAPE statistics.

Parameter	Subject (Gender, Height, Weight)	Strap	Reference	MAE	MAPE	Vest	Reference	MAE	MAPE
Average HR (bpm)	M 178/80	87	87	0.00	0.00%	95	88	7.00	7.95%
	M 177/70	119	120	1.00	0.83%	134	135	1.00	0.74%
	M 183/95	131	131	0.00	0.00%	141	127	14.00	11.02%
	F 167/74	136	136	0.00	0.00%	-	-	-	-
	M 193/115	134	134	0.00	0.00%	137	137	0.00	0.00%
	F 177/68	114	114	0.00	0.00%	107	106	1.00	0.94%
	M 172/76	98	98	0.00	0.00%	100	100	0.00	0.00%
	F 164/55	-	-	-	-	137	137	0.00	0.00%
	M 178/70	121	121	0.00	0.00%	112	112	0.00	0.00%
	F 154/51	145	145	0.00	0.00%	147	147	0.00	0.00%
	M 188/99	-	-	-	-	102	102	0.00	0.00%
	M 185/81	101	101	0.00	0.00%	114	92	22.00	23.91%
	M 181/74	131	131	0.00	0.00%	125	125	0.00	0.00%
	M 184/78	137	137	0.00	0.00%	143	144	1.00	0.69%
	M 181/74	108	108	0.00	0.00%	110	111	1.00	0.90%
	M 172/115	-	-	-	-	156	156	0.00	0.00%
	M 184/85	140	140	0.00	0.00%	148	148	0.00	0.00%
	F 176/67	128	128	0.00	0.00%	125	126	1.00	0.79%
	F 169/75	160	160	0.00	0.00%	152	152	0.00	0.00%
	M 174/72	128	128	0.00	0.00%	114	114	0.00	0.00%
	M 190/92	97	97	0.00	0.00%	99	100	1.00	1.00%
	M 177/93	126	126	0.00	0.00%	120	120	0.00	0.00%
	M 181/76	122	122	0.00	0.00%	136	136	0.00	0.00%
Mean	-	123.15	123.20	0.05	0.04%	125.18	123.41	2.23	2.18%
Energy Expenditure. Total (kcal)	M 178/80	94.40	98.98	4.58	4.63%	141.63	108.82	32.81	30.15%
	M 177/70	204.47	205.88	1.41	0.68%	241.76	246.46	4.70	1.91%
	M 183/95	213.98	215.71	1.73	0.80%	260.59	238.16	22.43	9.42%
	F 167/74	172.95	180.15	7.20	4.00%	-	-	-	-
	M 193/115	349.38	358.71	9.33	2.60%	357.56	371.34	13.78	3.71%
	F 177/68	113.84	113.62	0.22	0.19%	107.18	112.06	4.88	4.35%
	M 172/76	123.12	120.97	2.15	1.78%	115.30	115.95	0.65	0.56%
	F 162/58	-	-	-	-	-	-	-	-
	F 164/55	-	-	-	-	156.74	156.14	0.60	0.38%
	M 178/70	166.11	168.83	2.72	1.61%	155.18	157.46	2.28	1.45%
	F 154/51	149.00	149.93	0.93	0.62%	133.72	134.79	1.07	0.79%
	M 188/99	-	-	-	-	151.04	149.16	1.88	1.26%
	M 185/81	134.59	134.12	0.47	0.35%	190.08	109.47	80.61	73.64%
	M 181/74	242.03	243.45	1.42	0.58%	229.26	233.38	4.12	1.77%
	M 184/78	236.58	239.49	2.91	1.22%	226.36	234.46	8.10	3.45%
	M 181/74	157.84	152.67	5.17	3.39%	166.31	162.70	3.61	2.22%
	M 172/115	-	-	-	-	473.98	475.94	1.96	0.41%
	F 162/63	-	-	-	-	-	-	-	-
	M 184/85	295.42	296.21	0.79	0.27%	324.80	323.31	1.49	0.46%
	F 176/67	151.10	157.73	6.63	4.20%	153.35	153.98	0.63	0.41%
	F 169/75	256.15	255.17	0.98	0.38%	245.48	254.89	9.41	3.69%
	M 174/72	230.04	233.34	3.30	1.41%	199.91	200.73	0.82	0.41%
	M 190/92	141.85	143.02	1.17	0.82%	147.07	145.57	1.50	1.03%
	M 177/93	238.35	249.20	10.85	4.35%	226.92	237.19	10.27	4.33%
	M 181/76	210.04	210.40	0.36	0.17%	241.62	241.85	0.23	0.10%
Mean	-	194.06	196.38	3.22	1.70%	211.17	207.45	9.45	6.63%
EPOC Peak (ml/kg)	M 178/80	4.30	4.56	0.26	5.70%	9.61	4.92	4.69	95.33%
	M 177/70	21.64	22.14	0.50	2.26%	36.42	39.24	2.82	7.19%
	M 183/95	29.01	28.85	0.16	0.55%	40.90	29.54	11.36	38.46%
	F 167/74	36.86	40.51	3.65	9.01%	-	-	-	-
	M 193/115	32.50	35.20	2.70	7.67%	32.75	36.48	3.73	10.22%
	F 177/68	14.13	14.28	0.15	1.05%	11.30	11.74	0.44	3.75%
	M 172/76	5.59	5.69	0.10	1.76%	5.12	5.66	0.54	9.54%
	F 162/58	-	-	-	-	-	-	-	-
	F 164/55	-	-	-	-	43.71	44.00	0.29	0.66%
	M 178/70	20.05	21.01	0.96	4.57%	13.93	14.57	0.64	4.39%
	F 154/51	51.83	52.31	0.48	0.92%	52.94	54.07	1.13	2.09%
	M 188/99	-	-	-	-	9.45	9.17	0.28	3.05%
	M 185/81	5.55	5.49	0.06	1.09%	34.06	3.29	30.77	935.26%
	M 181/74	31.94	33.16	1.22	3.68%	23.39	24.37	0.98	4.02%
	M 184/78	36.74	37.54	0.80	2.13%	39.99	44.90	4.91	10.94%
	M 181/74	11.20	10.47	0.73	6.97%	9.52	10.81	1.29	11.93%
	M 172/115	-	-	-	-	123.98	125.11	1.13	0.90%
	F 162/63	-	-	-	-	-	-	-	-
	M 184/85	40.36	40.96	0.60	1.46%	52.49	53.75	1.26	2.34%
	F 176/67	19.51	22.43	2.92	13.02%	17.19	18.15	0.96	5.29%
	F 169/75	98.77	96.24	2.53	2.63%	72.07	83.49	11.42	13.68%
	M 174/72	24.71	25.41	0.70	2.75%	14.01	14.24	0.23	1.62%
	M 190/92	8.62	8.66	0.04	0.46%	9.66	10.47	0.81	7.74%
	M 177/93	25.67	28.59	2.92	10.21%	14.53	18.83	4.30	22.84%
	M 181/76	36.17	36.20	0.03	0.08%	51.46	51.49	0.03	0.06%
Mean		27.76	28.49	1.08	3.90%	32.66	32.20	3.82	54.15%
TRIMP (index)	M 178/80	13.00	13.00	0.00	0.00%	17.00	13.00	4.00	30.77%
	M 177/70	28.00	28.00	0.00	0.00%	37.00	37.00	0.00	0.00%
	M 183/95	25.00	25.00	0.00	0.00%	34.00	27.00	7.00	25.93%
	F 167/74	37.00	37.00	0.00	0.00%	-	-	-	-
	M 193/115	33.00	33.00	0.00	0.00%	34.00	34.00	0.00	0.00%
	F 177/68	17.00	17.00	0.00	0.00%	17.00	16.00	1.00	6.25%
	M 172/76	12.00	12.00	0.00	0.00%	11.00	11.00	0.00	0.00%
	F 162/58	-	-	-	-	-	-	-	-
	F 164/55	-	-	-	-	35.00	35.00	0.00	0.00%
	M 178/70	22.00	22.00	0.00	0.00%	19.00	19.00	0.00	0.00%
	F 154/51	36.00	36.00	0.00	0.00%	33.00	33.00	0.00	0.00%
	M 188/99	-	-	-	-	9.00	9.00	0.00	0.00%
	M 185/81	16.00	16.00	0.00	0.00%	29.00	13.00	16.00	123.08%
	M 181/74	28.00	28.00	0.00	0.00%	26.00	26.00	0.00	0.00%
	M 184/78	28.00	28.00	0.00	0.00%	30.00	30.00	0.00	0.00%
	M 181/74	13.00	13.00	0.00	0.00%	14.00	14.00	0.00	0.00%
	M 172/115	-	-	-	-	78.00	78.00	0.00	0.00%
	F 162/63	-	-	-	-	-	-	-	-
	M 184/85	32.00	32.00	0.00	0.00%	39.00	38.00	1.00	2.63%
	F 176/67	23.00	23.00	0.00	0.00%	22.00	22.00	0.00	0.00%
	F 169/75	58.00	58.00	0.00	0.00%	52.00	53.00	1.00	1.89%
	M 174/72	26.00	26.00	0.00	0.00%	20.00	20.00	0.00	0.00%
	M 190/92	12.00	13.00	1.00	7.69%	13.00	14.00	1.00	7.14%
	M 177/93	23.00	23.00	0.00	0.00%	20.00	20.00	0.00	0.00%
	M 181/76	30.00	30.00	0.00	0.00%	39.00	39.00	0.00	0.00%
Mean		25.60	25.65	0.05	0.38%	28.55	27.32	1.41	8.99%
